# Milestones in acute GVHD pathophysiology

**DOI:** 10.3389/fimmu.2022.1079708

**Published:** 2022-12-05

**Authors:** Gerard Socie, David Michonneau

**Affiliations:** ^1^ Université Paris Cité, Paris, France; ^2^ APHP, Hématologie Greffe, Hôpital Saint Louis, Paris, France; ^3^ INSERM UMR 976, Hôpital Saint Louis, Paris, France

**Keywords:** GvHD, history, acute, pathophysiology, milestones

## Abstract

In the past 65 years, over 25 000 referenced articles have been published on graft-versus-host disease (GVHD). Although this included clinically orientated papers or publications on chronic GVHD, the conservative estimate of scientific publications still contains several thousands of documents on the pathophysiology of acute GVHD. Thus, summarizing what we believe are prominent publications that can be considered milestones in our knowledge of this disease is a challenging and inherently biased task. Here we review from a historical perspective what can be regarded as publications that have made the field move forward. We also included several references of reviews on aspects we could not cover in detail.

## Introduction

It has been more than 65 years since the seminal description that mice exposed to lethal irradiation and received allogeneic splenocytes developed diarrhea and skin lesions and died (1, 2). Initially designated as a “secondary disease, “ this syndrome was indeed graft-versus-host disease (GVHD). Ten years later, Matheí and colleagues reported the first patient who underwent allogeneic bone marrow transplantation (BMT) who developed lethal GVHD (3). The outcomes of patients transplanted at this early age were poor [reviewed in (4)]. This mainly resulted from the poor knowledge of histocompatibility, the need for immunosuppressive drugs post-transplant, and advanced stage of the hematological malignancies.

**Figure 1:**
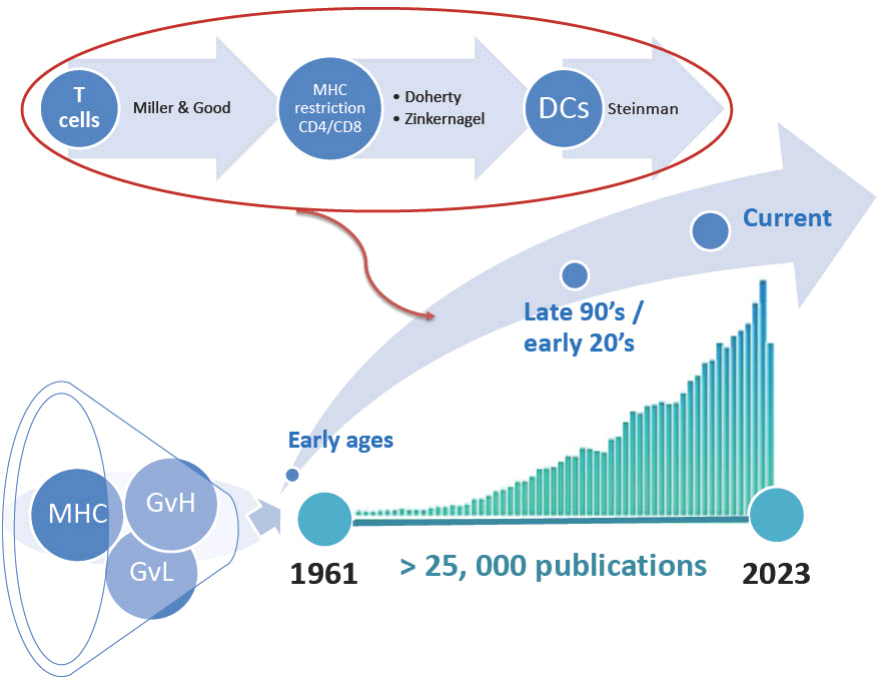
Referenced publications on graft-versus host disease (GVH). The diagram illustrates the number of publications referenced in PubMed as of October 2022. MHC, major histocompatibility complex; GvL, graft-versus-leukemia effect; DCs, Dendritic cells.

Thousands of patients have since been transplanted, and our understanding of the disease has improved. At the time of writing this perspective, we made a PubMed search on “GVHD included in the title or the abstract.” This resulted in over 25,000 referenced publications (**Figure 1**). Thus, when we naively accepted to write a review on a historical perspective of GVHD, we were stuck with the tough task of choosing among these 25 000 publications what we believed could be the milestones in the understanding GVHD pathophysiology. The obvious consequences of this unattainable task are the following: 1) we must drastically limit the number of papers to be cited, and: 2) this perspective is inherently associated with our own bias on what we believe are milestones! Thus, we deeply apologize for non-citing major articles from friends and colleagues in this manuscript and refer the general audience of this Journal to excellent reviews that cover this field more extensively [reviewed in (5–10)]. Finally, to keep the field of this perspective in a narrower ambition, we decided to restrict the subject to acute GVHD. Again, readers interested in chronic GVHD will find excellent reviews elsewhere [reviewed in (6, 11)].

## Classical steps of GVHD pathophysiology

Major milestones from the early ages of hematopoietic stem cell transplantation deserve first to be mentioned. Pioneers in the field included Van Bekkum, Thomas, and Santos [reviewed in (4, 12–14)]. In parallel, in this period, the major histocompatibility complexes (MHC) in the mice (15) and humans (16) were discovered. Similar advances were made in the MHC typing of dogs and later in non-human primates (17, 18). These early ages were also associated with a bloom in our knowledge of basic (and transplant) immunology that set the basis for developing our knowledge of GVHD (19, 20). But the foundation of our ongoing effort to understand GVHD should be attributed to Billingham, who described the three prerequisite mechanisms for developing GVHD (21, 22); “*the graft must contain immunologically competent cells; the recipient must express antigens that are not present in the transplant donor, and the recipient must be incapable of mounting an effective response to eliminating the transplanted cells*” [summarized by Ferrara in (5)].

In the late ‘70s, another piece of complexity was introduced with the advent of minor histocompatibility antigens (mi-Ha) in mice that led to GVHD but was prevented by the removal of T-lymphocytes from the graft (23). It then took 20 years before the role of mi-Ha was strongly suggested in humans (24), although the role of individual mi-Ha remains controversial (25).

In the past 60 years, our knowledge of acute GVHD has dramatically improved. Readers with interest might compare figures on the pathophysiology of acute GVHD published by Ferrara and Deeg in 1991 (26) to those published by Zeiser and Blazar (10) or Hill and coworkers (6) to get a sense of how much we improved (although much more science is still needed)!.

The biology of acute GVHD has been classically described by successive steps from conditioning-induced tissue damage to the induction phase, donor T-cell expansion, and recruitment of neutrophils and macrophages, to the effector stage and tissue damages (5). However, describing these stages from a historical perspective to describe what have been the significant milestones in acute GVHD pathophysiology poorly fit with this classical circle. In each of the following sections, we aimed to give credit to the first seminal papers and then discussed more recent reports to show how each step evolved during the time. Finally, we introduce in the last sections new aspects that help, in our view, the field to move forward.

## Initiation of the allogeneic immune response

### The conditioning-induced damages and their early consequences

In the clinical setting, pre-transplant conditioning regimens has long been associated with GVHD’s incidence and severity. In experimental models, Ferrara’s group also demonstrated increased severity of GVHD with increasing dose of total body irradiation (TBI). Increased GVHD was mediated by systemic increases in TNFα. TBI-induced damages increased the translocation of LPS, and LPS triggered excess TNFα alpha from macrophages primed by the GVH reaction as first described by Nestel and coworkers (27) and later confirmed by others (28, 29). T cells in irradiated allogeneic recipients lead to their rapid recruitment to nonlymphoid tissues, where they induce GVHD. But it was later elegantly demonstrated that delayed T-cell injection does not induce GVHD (30). These first pieces of evidence show that the gastrointestinal (GI) tract as a central role in the GVHD pathogenesis, which was then referred to as the “cytokine storm” (31). From a mechanistic point of view, it took another ten years to demonstrate that danger signals released upon cell damage after irradiation, namely ATP, when released from dying cells, increased CD80 and CD86 expression and initiate proinflammatory events, with STAT1, interferon-γ production, donor T cell expansion, and increased expression of the ATP’s receptorP2X (7) R (32). Finally, neutrophils increased conditioning-induced tissue damage caused, and increased GVHD-related mortality. This phenomenon was dependent on reactive oxygen species (ROS). In contrast, the transfer of neutrophils lacking Toll-like receptors led to reduced GVHD severity (33).

### Cytokines and chemokines

The first cytokines associated with GVHD-associated tissue lesions were TNFα and IL-1 (34, 35). These findings and other data led Antin and Ferrara to suggest that the recruitment and activation of mononuclear effector cells, which produce inflammatory cytokines, is critical for amplifying the systemic inflammatory response (36). These first publications suggested that GVHD is initiated by alloreactive type 1 T cells that secrete gamma-interferon (IFN-γ) which induces the production of other inflammatory cytokines (TNFα and IL-1). Used polarized type-2 T-cells (secreting IL-4 but not IFN-γ) led to decreased production of both IFN-γ and TNFα despite persistent pathological changes (37). Chemokine and chemokine receptor interactions and adhesion molecules have also been shown to play roles in effector cell migration in experimental GVHD models. A review in the early 20’s discussed the role of chemokines during experimental GVHD (38). However, even to date, it is hard to decipher the actual role of chemokines/chemokine receptors in the activation phase of GVHD, most probably because of the redundant nature of these molecules that recognize different receptors with receptors able to identify different ligands. Finally, intestinal commensal bacteria and uric acid contribute to Nlrp3 inflammasome-mediated IL-1β production after conditioning. Blockade of IL-1β or IL-1-R -/- dendritic cells (DCs) and T cells improved survival. IL-1β originated from different intestinal cells and affected DCs and TH17 cells (39).

### T-cell migration into lymphoid organs

The migration and homing of cells in GVHD *in vivo* was assessed by imaging technics using enhanced green fluorescence protein transgenic-, or luciferase-labeled, allogeneic T cells (40, 41). These *in vivo* experiments demonstrated an early migration of allogeneic cells first to peripheral lymphoid organs, and then to GVHD target organs. A very early expansion of T-cells in Peyer’s patches was of interest. Early in the 20s, it was demonstrated that naive T cells migrate to secondary lymphoid organs where they underwent activation by antigens and acquired the ability to home to nonlymphoid sites. Activated effector/memory T cells migrate preferentially to tissues where the antigen was first encountered. Essential receptors for intestinal homing α4β7, the receptor for the gut-associated chemokine CCL25, were described. A paper by Von Adrian’s group (outside the setting of transplantation) showed that imprinting toward the GI tract is mediated by dendritic cells (DCs) from Peyer’s patches (42). In the background of GVHD, Murai et al. (43) then demonstrated that Peyer’s patches are an essential site in initiating murine acute GVHD [although this essentiality was, after that, not confirmed (44)].

## Antigen presentation and costimulation

Allogeneic T cells stimulation was mysterious till the end of the 90s. In a seminal paper, Shlomchik and Emerson elegantly demonstrated in CD8-mediated, multiple mi-Ha mismatched, models that (APCs) only host-derived antigen-presenting cells (APCs) initiated graft versus host disease (45). Subsequently, Teshima and Ferrara (46) found that, in CD4-mediated models, acute GvHD does not require alloantigen expression on host target epithelium, in a CD4-mediated model. Then, it was demonstrated that although host APCs are mandatory to initiate acute GVHD, donor APCs are required to perpetuate it (47). DCs are not only critical for GVHD induction, DCs were then shown to induce organ tropism through the induction of organ-specific homing molecules T-cells. Gut-derived DCs induced alloreactive gut-homing T-cells leading to increased GVHD severity (48). MHC class II antigen presentation by intestinal epithelial cells (IECs) was then demonstrated within the ileum at steady state. IEC-specific deletion of MHC class II prevented lethal GVHD in the GI tract, and the development of GVHD required IFNγ secretion by lamina propria lymphocytes (49). Finally, in the colon donor CD103(+)CD11b (–) DCs migrate to mesenteric lymph nodes, also leading to gut-homing signatures on donor T cells, leading to GI tract GVHD (50).

T-cell activation needs costimulation that has also been extensively studied within the context of GVHD from a therapeutical perspective. In 1994, Blazar et al. reported that blockade of the CD28/CTLA4: CD80-CD86 interaction reduces lethal GVHD in a major mismatched mice model (51). This was confirmed later in the non-human primate model (NHP) (52, 53) and had recently shown promising results in human GVHD (54). Blockade of the CD40/CD40-ligand ICOS and OX40-ligand has also been reported both in rodents and in NHP models (55–59). Increased GVHD in recipients of B7-H3(-/-) Treg-depleted grafts was also described (60). The role of ICOSL+ DCs was also recently explored both in humans and in experimental models. Increased numbers of ICOSL(+) plasmacytoid DCs (p-DCs), which correlate with CD146+CCR5+ T cell, were found in patients with GI GVHD. ICOSL-deficient mice had reduced GVHD, and prophylaxis with a dual ICOS/CD28 antagonist prevented acute GVHD (61).

Langerhans cells (LCs) act as network in the epidermis of the skin. Using an inducible depletion of host LCs experimental model, Bennet et al. found reduced GVHD when LCs were absent. However, LCs were not required either for CD8 T-cell activation or homing to the epidermis. However, LCs were necessary for licensing IFN-γ secreting donor CD8 cells in the epidermis to induce epithelial injury (62).

## Effector- and regulatory-T cells

Early studies showed that both the Fas/Fas-L and perforin/granzyme cytotoxicity were important in inducing GVHD lesions (63–65). Then the development of flow cytometry allowed us to decipher which T-lymphocyte subset mediates GVHD. The landmark papers by Anderson et al. (66, 67) reported that memory CD4 cells induced neither clinical nor histologic GVHD and argued subsequently against the hypothesis that T effector memory cells are unable to generate GVHD because of inefficient trafficking to lymphoid organs. *In vivo* analyses of the early event in murine models also support the leading role of naïve T-cells (41). These results prompted considering naïve T-cell depletion as a clinical tool to decrease GVHD (68). Of special interest, however, is the paper by Emerson’s group in 2005 that describes a CD44(lo)CD62L(hi)CD8(+) T cell subset that generate and sustain GVHD with self-renewing capacity. These cells are Sca-1+, CD122+, and Bcl-2+ and induce GVHD in secondary recipients (69, 70). This paper is, in fact, the first description of the T-stem cell memory subset and was described in GVHD! Subsequently, with a growing focus on gastrointestinal (GI) GVHD, the role of TH17/TC17 has been the focus of research (71, 72). Finally, thanks to the development of molecular immunology, the activity of JAK 1/2 inhibition blockade was proven both in experimental studies and in humans (69), leading to a successful randomized clinical trial in steroid-resistant acute GVHD (73).

The allogeneic reaction is, however, subjected to cell-based regulation, primarily by regulatory T-cells (Treg). The field of Treg has been initially the subject of controversies, as nicely reviewed by Sakagushi (74). In GVHD three groups almost simultaneously published data in the early ‘20s that convincingly demonstrated that Tregs inhibit GVHD (75–78) while preserving the graft-versus-leukemia effect (79).

## The GI tract and the microbiota in GVHD

Although included in the classical Ferrara’s circled schema of GVHD’s pathogenesis (31, 80), it seems us that the GI tract and its relationship with the microbiome deserve special attention in this milestones’ paper because significant recent advances in the knowledge of the dialogue between the microbiome and the intestine in the GVHD process [reviewed in (81)].

During the past ten years, more careful attention has been paid to IEC subsets (i.e., the targets) than on effectors (i.e., T-cell subsets and regulators). Somewhat arbitrary, we will first allude to IEC and second to microbiota, although, obviously, these aspects are interrelated.

As already stated GI tract damages are critical for the amplification of GVHD (31). Intestinal stem cells (ISCs) regenerate the intestinal epithelium after injury. Ten years ago, Teshima’s group demonstrated that the pretransplant conditioning regimen damaged ISCs, and GVHD markedly inhibited ISC recovery. The Wnt agonist R-spondin1 (R-Spo1) protected ISC and increased intestinal epithelium healing (82). Almost simultaneously, it was demonstrated that IL-22 deficient recipients have increased tissue damage and mortality and that ISCs expressed the IL-22 receptor. Intestinal IL-22 was produced by recipient-derived innate lymphoid cells (ILCs). This IL-22 deficiency in the recipient led to increased crypt apoptosis, depletion of ISCs, and loss of epithelial integrity (83). Outside the field of GVHD, it was then reported that Lgr5+ ISCs are the target of activated T cells while quiescent stem cells from the hair follicle were resistant to T cell killing. Immune evasion of the quiescent stem cells resulted from downregulation of the antigen presentation machinery (84). But a broad image of tissue damage was provided by 3D imaging to analyze T-cell localization by Hanash’s group (85). They nicely reported that T-cell recruitment to the basal layer of the crypt leads to direct T cell engagement with MHC+ ISCs and their subsequent loss.

Another cell subset, Paneth cells (PCs), was also studied, and it was demonstrated that GVHD disrupts the intestinal microbiome by inhibiting Paneth cell production of α-defensins (86) and restoration of the gut microbiota by R-Spo1 or recombinant α-defensin in mice (84), thus linking R-Spo1 protection of ISC damage (82, 87) with the intestinal microbiota. Recently, Zeiser’s group explored another approach that aims at protecting and regenerating Paneth cells (PCs) and intestinal stem cells (ISCs). Glucagon-like-peptide-2 (GLP-2), an enteroendocrine hormone produce by intestinal L cells, is decreased both in mice and in patients developing GVHD. Treatment with the GLP-2 agonist, teduglutide, reduced de GVHD and steroid-refractory GVHD by promoting the regeneration of PCs and ISCs (88).

Finally, after ISCs, PCs, and L cells GI-cell subsets, the role of goblet cells was explored in GI GVHD. Intestinal goblet cells form the mucus layers, which serves as a barrier between the gut microbiota and the host tissues. Goblet cell loss is one of the histologic features of GVHD. This leads to a disruption of the mucus layer and increased bacterial translocation. Pretransplant administration of interleukin-25 (IL-25) protected goblet cells from GVHD, prevented bacterial translocation, reduced IFN-γ and IL-6 plasma levels, and ameliorated GVHD. The protective role of IL-25 depended on Lypd8, an enterocyte-derived antimicrobial molecule that suppresses the motility of flagellated bacteria (89).

From the above-reviewed data, it was obvious that the IECs should be studied in the context of their environment, i.e., the microbiota, a concept already brought by Van Bekkum in the early ages of BMT when he described that there is a “*Mitigation of secondary disease of allogeneic mouse radiation chimeras by modification of the intestinal microflora*” (14). In our current understanding, the seminal paper was published ten years ago by Jenq and coworkers (90). In this paper, authors demonstrated in murine and human recipients that intestinal inflammation secondary to GVHD is associated with significant intestinal dysbiosis with loss of overall diversity and expansion of Lactobacillales and loss of Clostridiales. This dysbiosis early after allogeneic BMT was a risk factor for subsequent GVHD. The increased GVHD-related mortality was associated with broad-spectrum antibiotic use after BMT in humans and mice (91). This was confirmed in more than 1000 patients, where studies on more than 8000 fecal samples disclosed patterns of microbiota disruption characterized by loss of diversity and domination by single taxa. Higher diversity was associated with a lower risk of death overall and death attributable to GVHD (92). Thus, while broad-spectrum antibiotics increase the risk of intestinal GVHD, the mechanisms remained elusive. In a recent study, Jenq’s laboratory found that treatment with meropenem aggravated colonic GVHD in mice *via* the expansion of Bacteroides thetaiotaomicron (BT) which can degrade dietary polysaccharides and host mucin glycans. BT in meropenem-treated mice upregulated enzymes involved in the degradation of glycans within the mucus leading to thinning of the colonic mucus layer and decreased levels of xylose in the lumen. Finally, oral xylose supplementation prevented thinning of the colonic mucus layer (93).

Several studies aimed to decipher the mechanisms of the crosstalk between GVHD and the microbiota from these experimental and clinical data. In one study, recipients lacking the ability to generate or signal IL-17 develop hyper-acute intestinal GVHD. When authors cohoused wild-type (WT) with IL-17RA and IL-17R-deficient mice, they found increased GVHD severity in WT mice, and the gut microbiome of WT mice shifted toward that of the IL-17RA/C mice (94). From a microbiological point of view, a high incidence of enterococcal expansion was then described, which was associated with GVHD and mortality in humans. Enterococcus also expanded in the mouse GI tract after transplantation. Enterococcus growth depends on Lactose, and lactose depletion attenuates Enterococcus outgrowth and reduces the severity of GVHD in mice (95). To decipher the impact of intestinal microbiota on microbial metabolites in GVHD, in a major paper by Reddy’s group identified alterations in microbiota-derived short-chain fatty acids (SCFAs) and found that only one SCFA, butyrate, was decreased in the intestinal tissue. The reduced butyrate in IECs resulted in decreased histone acetylation, which was restored after the administration of butyrate. Butyrate restoration improved IEC junctional integrity, reduced apoptosis, and mitigated GVHD (96). The same group then demonstrated that the metabolite sensor G-protein-coupled receptor 43 (GPR43) was critical for the protective effects of butyrate and require GPR43-mediated ERK phosphorylation and activation of the NLRP3 inflammasome in recipients’ target tissues (97).

## GVHD pathophysiology: What’s next?

From the above-summarized data, we would finally put into perspective other ways of thinking about GVHD pathophysiology.

### Tissue tolerance and compartmentalization of acute GVHD

Polly Matzinger first described the concept of tissue tolerance in 2011 (98). Far outside the field of GVHD, she studied the perspective on how the immune response is regulated. Rather than describing how immune cells are turned on and off, she focused on the second significant aspect of an immune response: the control of effector class. She suggested that the immune response is “*primarily tailored to fit the tissue in which the response occurs*”. More recently, Wu and Reddy (99) nicely discussed tissue damages in the setting of GVHD and underlined that acute GVHD had been mostly studied through on the aspect on how immune cells generate tolerance. However, tissue-intrinsic factors might contribute to the regulation of acute GVHD severity. They thus introduced the concept of “tissue tolerance” with the aim of integrating targeted tissue repair and regeneration, which may mitigate the severity of acute GVHD. Such a concept has also been advocated by Chakraverty and Teshima more recently (100).

More broadly, the compartmentalization of the allogeneic reaction may warrant further attention. Whether GVHD and GVL behave equally among different tissues remain unclear. An *in vivo* experimental study indeed showed that CTLs exhibited different cytotoxic function in distinct tissues after transplantation, being highest in the liver and lowest in lymph nodes. These differences came from the impact of the microenvironment, through differential PD-1 ligand expression. PD-1 blockade restored CTL sensitivity to antigens and killing in lymph nodes (101).

### Rebooting immunology in GVHD pathophysiology

As we reviewed elsewhere in the issue (Michonneau & Socie) impressive bloom of new technologies has emerged in the field of biology in the past few years, including single-cell technologies that allow studying pathophysiology at the genomic, epigenetic, transcriptomic, and protein levels. These have obvious consequences for deciphering in depth the pathophysiology mechanisms of GVHD. Most of the data described here come from experimental mice models of acute GVHD. We have already described elsewhere how important these models have been operative in deciphering the mechanisms of GVHD and have been able to set the stage for (eventually) successful clinical trials (8). As nicely reviewed by Brodin and Davis (102), conceptual and technological progress in human immunology has been impressive over the past few years. In particular single-cell technologies which allow in depth analyses with small quantities of human material allow now to study in far more depth immunological mechanisms in humans, and to integrate the impact of genetic or environmental associated factors.

The first study that opened the gate in human GVHD was that of Claude Perreault’s group in 2007 (103). In this seminal paper, authors asked if transcriptional profiles may identify “dangerous” donors more prone to mount a strong allogeneic reaction and, consequently, more likely to elicit GVHD. They measured gene expression of purified CD4(+) and CD8(+) T cells from 50 donors and found that pre-transplant profiling segregates donors whose recipient suffered from GVHD or not. This phenomenon is under polygenic control and coordinated by genes that regulate transforming growth factor-beta signaling and cell proliferation. Another step forward was then achieved in the NHP model by Leslie Kean’s group which quantified gene expression profile of non-human primate (NHP) T cells during acute GVHD. They identified signatures specific for alloreactive T cells and determined the impact of both mTOR and calcineurin inhibition on GVHD and discovered a druggable target aurora kinase A (104). Using high throughput plasma metabolomic in two cohorts of genotypically HLA-identical related pairs we recently reported that the metabolomic profiles markedly differed between recipients and donors. In particular, at GvHD onset, we identified significant variation in microbiota-derived metabolites, in particular of aryl hydrocarbon receptor (AhR) ligands and bile acids. We thus suspected that lower microbiota-derived AhR ligands may limit indoleamine 2,3-dioxygenase induction and thus influence alloreactivity (105).

Most recently, 3 groups shed new light on T-resident memory (Trm) T-cells in NHP and humans. In the NHP study, authors analyzed the transcriptomic of circulating, emigrating or already existing tissue-resident T cells in aGVHD target organs. Combining intravascular staining technique with single-cell RNA sequencing they demonstrate that T cell infiltration of the GI by donor CD8+ T cells was associated with an activated/cytotoxic phenotype and provided a canonical Trm signature (106). In humans, three other studies also pointed out that skin contains a population of host derived CD69+ αβ Trm that persist and remain functionally competent for years after BMT. Single-cell RNA sequencing revealed low expression of genes encoding tissue egress by Trm in the skin. However, these Trm T-cells are characterized by the expression of molecules that favor tissue retention and display stem cell markers (107–109). Finally, most studies focused on failure (i.e., GVHD), but none paid attention to operational tolerance without GVHD. In a recent study, we studied who underwent transplantation from HLA-identical siblings. Primary tolerance was associated with a long-lasting reshaping of the recipient’s immune system toward a regulatory T cell phenotype, and decreased T cell activation, proliferation, and migration. When we compared individuals with operational tolerance and nontolerant recipients, we observed alterations centered on CD38+-activated T and B cells in nontolerant patients. In tolerant patients, cell subsets with regulatory functions were prominent and RNA sequencing demonstrated several modifications, particularly with overexpression of the ectoenzyme NT5E (encoding CD73). Metabolomic analyses suggested a central role of androgens in operational tolerance. These data were confirmed using an integrative approach including the 3 omics tools and clinical parameters providing a resource to study operational tolerance in humans (110).

It is our own bias to believe that modern tools, including single cell multi-omics, both in experimental transplantation and in the human setting open a new area in the study of the pathophysiology of acute GVHD. Furthermore, recent development in tissue imaging (including mass cytometry) and spatial transcriptomics will allow the target organ. We might thus be faced with new studies where in-depth description of affected organs in human can lead to mechanistic experiments in the mice system.

## Author contributions

All authors contributed to the article and approved the submitted version.

## Acknowledgments

I (GS) would like to sincerely thank my friends and worldwide recognized experts in the immune biology of GVHD. They kindly agreed to give me their own top 10 references which they believe to be the milestones in this topic: Bruce Blazar, Jamy Ferrara, Pavan Reddy, Jerry Ritz, Takanori Teshima, Marcel Van Den Brinck, Robert Zeiser. Special dedication also to Claude Perreault, whose work inspired me to study the immune biology of allogeneic transplantation in humans.

## Conflict of interest

The authors declare that the research was conducted in the absence of any commercial or financial relationships that could be construed as a potential conflict of interest.

## Publisher’s note

All claims expressed in this article are solely those of the authors and do not necessarily represent those of their affiliated organizations, or those of the publisher, the editors and the reviewers. Any product that may be evaluated in this article, or claim that may be made by its manufacturer, is not guaranteed or endorsed by the publisher.
